# Coastal Biotechnology: Facing the Global and the Regional Changes

**DOI:** 10.1155/2014/191480

**Published:** 2014-04-17

**Authors:** Song Qin, Wei Zhang, Hanzhi Lin

**Affiliations:** ^1^Yantai Institute of Coastal Zone Research, Chinese Academy of Sciences, Yantai 264003, China; ^2^Flinders Centre for Marine Bioproducts Development, Flinders University, Adelaide, SA 5042, Australia; ^3^Environmental Biophysics and Molecular Ecology Program, Institute of Marine and Coastal Sciences, Rutgers University, New Brunswick, NJ 08901, USA

Coastal biotechnology is developing fast with exciting achievements being realized in biological, chemical, and environmental sciences and technologies. The aim of the 1st International Conference on Coastal Biotechnology (1st ICCB) on “Coastal Biotechnology for Sustainable Development,” which was successfully held in the Australian winter of 2012 in Adelaide, Australia, was to bring together the international community working on coastal biotechnology issues, to help strengthen connections in this highly interdisciplinary field, and to inspire new research and development from the scientific and industrial sectors.

The 1st ICCB had two themes: “the changing distribution pattern of coastal bioresources” and “coastal biotechnology: facing the global and regional changes.” Bioresources are the essence of biotechnology. We first need to understand the biodiversity of these bioresources before we choose the techniques needed to use them to our advantage. Products and industries that emerge from coastal biotechnologies influence research and the development of technologies and policies. We are living in a world with rapid global changes—both naturally and socially. Those changes could alter the patterns of biodiversity especially in marine and coastal environments. How we address the global questions of food, fuel, population, environment, and so forth with sustainable developments of coastal bioresources is an issue not only for marine biologists, but also for all people of insight both from academia and industry.

This special issue is devoted to coastal biotechnology, containing 7 papers that pertain to our goals in the ICCB conference. Papers from L. Ding et al., R. Xing et al., and X. Guan et al. are on seaweeds (marine macroalgae); the ones from Z. Liu et al and Y. Alkhamis et al. are on microalgae; the one from L. Li et al. focuses on halophyte; and the one from M. A. Hakim et al. is on coastal salt-tolerant crop, rice. These 7 papers are as follows: “*Cloning and expression of a cytosolic HSP90 gene in Chlorella vulgaris*” by Z. Liu et al.; “*Biochemical and anatomical changes and yield reduction in rice (Oryza sativa L.) under varied salinity regimes*,” by M. A. Hakim et al.; “*Cultivation of Isochrysis galbana in phototrophic, heterotrophic, and mixotrophic conditions*,” by Y. Alkhamis and J. G. Qin; “*Photosystem II photochemistry and phycobiliprotein of the red algae Kappaphycus alvarezii and their implications for light adaptation*,” by X. Guan et al.; “*Extraction and separation of fucoidan from Laminaria japonica with chitosan as extractant*,” by R. Xing et al.; “*Pyrolytic and kinetic analysis of two coastal plant species: Artemisia annua and Chenopodium glaucum*,” by L. Li et al.; and “*Effects of seawater salinity and temperature on growth and pigment contents in Hypnea cervicornis J. Agardh (Gigartinales, Rhodophyta)*,” by L. Ding et al.

The inspiration of this special issue has grown from the contributions from the combined conferences ICCB and the 8th Asia Pacific Conference on Algal Biotechnology (APCAB 2012). APCAB meetings date back to more than 20 years ago when the first was held in Malaysia in 1992. With its development over the years, APCAB is regarded as the major international forum in algal biotechnology R&D field, serving the Asia-Pacific region, offering not only an examination of achievements in algal biotechnology, but also a platform for discussing the roadmap for future developments.

